# Pyroptosis -related potential diagnostic biomarkers in steroid-induced osteonecrosis of the femoral head

**DOI:** 10.1186/s12891-023-06729-8

**Published:** 2023-07-25

**Authors:** Jin-Lian Chai, Bo-Wen Lu, Hai-Tao Du, Ming-Tao Wen, Xue-Zhen Liang, Ping Wang

**Affiliations:** 1 College of Pharmacy, Shandong University of Traditional Chinese Medicine, Jinan, 250355 Shandong China; 2 College of Traditional Chinese Medicine, Shandong University of Traditional Chinese Medicine, Jinan, 250355 Shandong China; 3 Shandong Provincial Research Institute of Traditional Chinese Medicine, Yanzi Shanxi Road No.7, Jinan, 250014 Shandong China; 4 First College of Clinical Medicine, Shandong University of Traditional Chinese Medicine, 16369 Jingshi Road, Shandong 250355 Jinan, China; 5 Orthopaedic Microsurgery, Affiliated Hospital of Shandong University of Traditional Chinese Medicine, Jinan, 250014 Shandong China

**Keywords:** Steroid induced osteonecrosis of the femoral head, Pyroptosis, Bioinformatics analysis, Gene expression omnibus, Diagnostic biomarkers

## Abstract

**Purpose:**

Steroid-induced necrosis of the femoral head (SONFH) is a refractory orthopedic hip disease occurring in young and middle-aged people, with glucocorticoids being the most common cause. Previous experimental studies have shown that cell pyroptosis may be involved in the pathological process of SONFH, but its pathogenesis in SONFH is still unclear. This study aims to screen and validate potential pyroptosis-related genes in SONFH diagnosis by bioinformatics analysis to further elucidate the mechanism of pyroptosis in SONFH.

**Methods:**

There were 33 pyroptosis-related genes obtained from the prior reviews. The mRNA expression was downloaded from GSE123568 dataset in the Gene Expression Omnibus (GEO) database, including 10 non-SONFH (following steroid administration) samples and 30 SONFH samples. The pyroptosis-related differentially expressed genes involved in SONFH were identified with “affy” and “limma” R package by intersecting the GSE123568 dataset with pyroptosis genes. In addition, Gene Ontology (GO) and Kyoto Encyclopedia of Genes and Genomes (KEGG) pathway enrichment analyses of the pyroptosis-related differentially expressed genes involved in SONFH were conducted by “clusterProfiler” R package and visualized by “GOplot” R package. Then, the correlations between the expression levels of the pyroptosis-related differentially expressed genes involved in SONFH were confirmed with “corrplot” R package. Moreover, the protein–protein interaction (PPI) network was analysed by using GeneMANIA database. Next, The ROC curve of pyroptosis-related differentially expressed genes were analyzed by “pROC” R package.

**Results:**

A total of 10 pyroptosis-related differentially expressed genes were identified between the peripheral blood samples of SONFH patients and non-SONFH patients based on the defined criteria, including 20 upregulated genes and 10 downregulated genes. The GO and KEGG pathway enrichment analyses revealed that these 10 pyroptosis-related differentially expressed genes involved in SONFH were particularly enriched in cysteine-type endopeptidase activity involved in apoptotic process, positive regulation of interleukin-1 beta secretion and NOD-like receptor signaling pathway. Correlation analysis revealed significant correlations among the 10 differentially expressed pyroptosis-related genes involved in SONFH. The PPI results demonstrated that the 10 pyroptosis-related differentially expressed genes interacted with each other. Compared to non-SONFH samples, these pyroptosis-related differentially expressed genes had good predictive diagnostic efficacy (AUC = 1.000, CI = 1.000–1.000) in the SONFH samples, and NLRP1 had the highest diagnostic value (AUC: 0.953) in the SONFH samples.

**Conclusions:**

There were 10 potential pyroptosis-related differentially expressed genes involved in SONFH were identified via bioinformatics analysis, which might serve as potential diagnostic biomarkers because they regulated pyroptosis. These results expand the understanding of SONFH associated with pyroptosis and provide new insights to further explore the mechanism of action and diagnosis of pyroptosis associated in SONFH.

## Introduction

Steroid induced osteonecrosis of the femoral head (SONFH) is a chronic refractory orthopaedic disease, which has extremely high disability rate [1, 2]. SONFH seriously threaten human health, especially in young and middle-aged men aged 30 to 50. If not treated promptly and effectively, about 80% of SONFH patients would evolve into femoral head collapse within 1 to 4 years, causing a heavy social and economic burden [3, 4]. Due to the lack of effective screening tools and the difficulties in early diagnosis, many patients are already in the middle to late stage the onset of the corresponding symptoms and at the time of X-ray diagnosis. The main current treatments for SONFH are medication, hip-conserving surgery, and hip replacement surgery. Patients may need to face multiple revision total hip surgeries due to the presence of dislocation, aseptic loosening, infection, and limitations on the service life of the prosthesis. Therefore, reliable new predictive and prognostic models of SONFH are urgently needed for early diagnosis to make targeted therapy more feasible and achieve early treatment.

Pyroptosis, a novel pattern of non-apoptotic programmed cell death due to cellular inflammatory necrosis [5–7], which the morphological features are swelling of cells, forming pyroptotic bodies, cell membrane rupture and releasing inflammatory cytokines. Pyroptosis was widely found in eukaryotic cell organisms and was a self-protective mode of cells against external damage, but excessive activation could lead to damage. A large number of experimental studies have shown that pyroptosis was closely related to the development of diabetes mellitus, rheumatoid arthritis, ankylosing spondylitis, osteomyelitis, infection, Parkinson's disease, tumor and other diseases [8–14]. The extensive use of glucocorticoids could activate bone marrow mesenchymal stem cells (BMSC) pyroptosis, which was closely related to the development of SONFH [15]. The cellular pyroptosis pathway was a multifactorial and multi-level and complex regulatory process [16] Wang et al. showed that the expression of NLRP3 and caspase-1 were increased in human BMSCs treated with lipopolysaccharide (LPS), partly suggesting a pyroptosis in BMSCs [17]. Chen Y et al. reported that the NLRP3 inflammasome could mediate cell pyroptosis in BMSCs and triggered the imbalance in osteoblasts and osteoclast differentiation, which might play a critical role in the development of SONFH [12]. As demonstrated by Shahzad K et al., the caspase-1-dependent inflammasome activation occupies an important role in the development and development of SONFH [16]. Although many scholars at home and abroad have revealed the role of different types of pyroptosis in SONFH, the specific regulatory mechanism of pyroptosis in SONFH is still unclear, and the specific relationship between pyroptosis and SONFH needs further study. To date, there have been no bioinformatics studies on the genes and pathogenesis of SONFH associated with pyroptosis. A large sample of patient data helped to determine the adapted disease stage and prognostic value of therapeutic interventions, and Zhang Y et al. performed a genome-wide analysis of human peripheral serum from SONFH patients and healthy individuals and uploaded the data to the dataset GSE123568 in the National Center for Biotechnology Information Gene Expression Omnibus database (NCBI GEO). Therefore, we proposed to conduct systematic studies using bioinformatics and experimental validation means to determine the expression levels of cellular pyroptosis-related genes in normal and SONFH tissues, to explore the prognostic value of these genes,and understand the pathogenesis of pyroptosis in SONFH, and provide potential biomarkers for the clinical diagnosis and treatment of SONFH.

## Materials and methods

### Pyroptosis -related gene datasets and microarray data

The 33 pyroptosis-related genes were extracted from prior reviews [18]. We obtained the mRNA dataset of 10 non-SONFH (following steroid administration) samples and 30 SONFH samples from NCBI GEO (https://www.ncbi.nlm.nih.gov/geo/, ID: GSE123568).

### Differentially expressed analysis of pyroptosis-related genes in SONFH

The “affy” and “limma” R package were used to identify the pyroptosis-related differentially expressed genes between non-SONFH samples and SONFH samples in GSE123568. The screening criterion as an adjusted *P*-value < 0.01 and |log2 fold change (FC)|> log_2_1.5. Additionally, the results were computed and visualized in the heatmaps and volcano plots by “pheatmap” and “ggplot2” R package.

### Functional enrichment analysis of pyroptosis-related differentially expressed genes in SONFH

Based on these pyroptosis-related differentially expressed genes in SONFH, Gene Ontology (GO) [19] and Kyoto Encyclopedia of Genes and Genomes (KEGG) [20] pathway enrichment analysis were carried out by “clusterProfiler” R package and visualized by “GOplot” R package. The GO enrichment analysis mainly included biological process (BP), cellular composition (CC), and molecular function (MF). The statistically significant difference of enriched terms was filtered according to specific criteria of an adjusted *P*-value < 0.05.

### Correlation analysis of the pyroptosis-related differentially expressed genes in SONFH

To explore the intrinsic relationship among these pyroptosis-related differentially expressed genes in SONFH, the Spearman correlation coefficient among each gene were calculated by the “corrplot” R package.

### Construction of PPI network of the pyroptosis-related differentially expressed genes in SONFH

To gain more insight into the in-depth relationships of pyroptosis-related differentially expressed genes in SONFH, a PPI network was predicted and constructed by the GeneMANIA database (http://www.genemania.org), [21] and which was constructed and visualized by Cytoscape software (version: 3.7.2, http://cyto-scape.org/). The minimum required interaction score for the PPI analysis was set at in a combined score >  = 0.4. The nodes represented genes, and the edges represented the interactive relations between the genes. and the isolated nodes were discarded.

### ROC curve analysis of the pyroptosis-related differentially expressed genes in SONFH

The “pROC” R package were employed to perform a ROC curve analysis of these pyroptosis-related differentially expressed genes in SONFH. The area under the curve (AUC) was calculated to analyse the intrinsic effectiveness of diagnostic tests, which was an indicator combining sensitivity and specificity between 0.5 and 1. The closer the AUC was to 1, the better the diagnosis was. The AUC had low accuracy at 0.5–0.7, and moderate accuracy at 0.7–0.9, and high accuracy above 0.9.

### Statistical analysis

All statistical analysis was accomplished with R software (version 4.0.2) in this study. Gene expression levels of samples in GSE123568 were employed by the Kruskal–Wallis test. The data are shown as means ± standard deviation. Independent-sample t tests were used to compare means between two different groups. Fisher's exact test was used to compare the incidence of disease between two different groups. Statistical analysis was conducted using GraphPad Prism (Version 9.0.0). A threshold for statistical significance was set as *P* < 0.05.

## Results

### Pyroptosis-related differentially expressed genes in SONFH

A total of 33 non-duplicated genes were excavated referring to the article of Ye et al., and a total of 31 expression of pyroptosis-related genes were intersected them with GSE123568. There were 10 pyroptosis-related differentially expressed genes were identified based on the defined criteria of an adjusted *P*-value < 0.05 and |logFC|> log_2_1.5, including 9 up-regulated genes and 1 down-regulated genes in SONFH samples compared to non-SONFH Samples (following steroid administration) in Table 1.

**Table 1:** The 10 pyroptosis-related differentially expressed genes in SONFH samples compared to non-SONFH samples

UniProtKB	Name	Symbol	logFC	*P*.Value	adj.P.Val
P29466	Caspase-1	CASP1	0.972	6.742E-05	0.000934
P49662	Caspase-4	CASP4	0.825	0.0001483	0.00167
P51878	Caspase-5	CASP5	0.835	0.00182	0.00979
P08246	Neutrophil elastase	ELANE	-1.164	0.00680	0.0241
P01584	Interleukin-1 beta	IL1B	0.735	0.00163	0.00906
Q9C000	NACHT, LRR and PYD domains-containing protein 1	NLRP1	1.120	1.243E-09	4.201E-07
Q96P20	NACHT, LRR and PYD domains-containing protein 3	NLRP3	0.701	1.743E-05	0.000364
Q9HC29	Nucleotide-binding oligomerization domain-containing protein 2	NOD2	0.893	0.00123	0.00749
Q9ULZ3	Apoptosis-associated speck-like protein containing a CARD	PYCARD	0.740	4.598E-05	0.000717
Q99590	Protein SCAF11	SCAF11	0.596	3.752E-05	0.000615

**Fig. 1:**
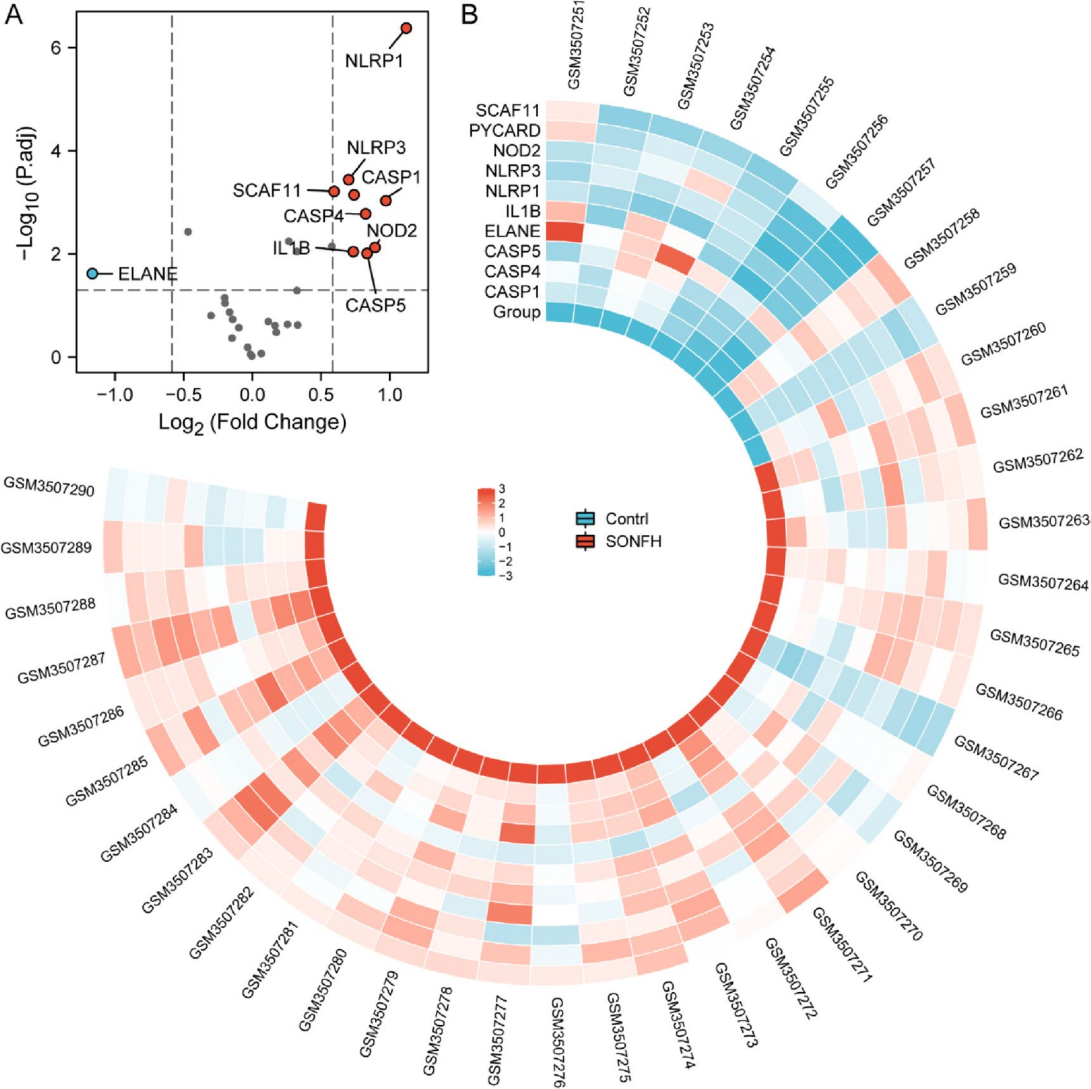
Pyroptosis-related differentially expressed genes in SONFH and non-SONFH samples. **A** Volcano plot of the pyroptosis-related genes intersected with GSE123568. The red dots represent the significantly up-regulated genes and the blue dots indicate the significantly down-regulated genes. **C** Circo heatmap of the 10 pyroptosis-related differentially expressed genes in SONFH and non-SONFH samples

**Fig. 2:**
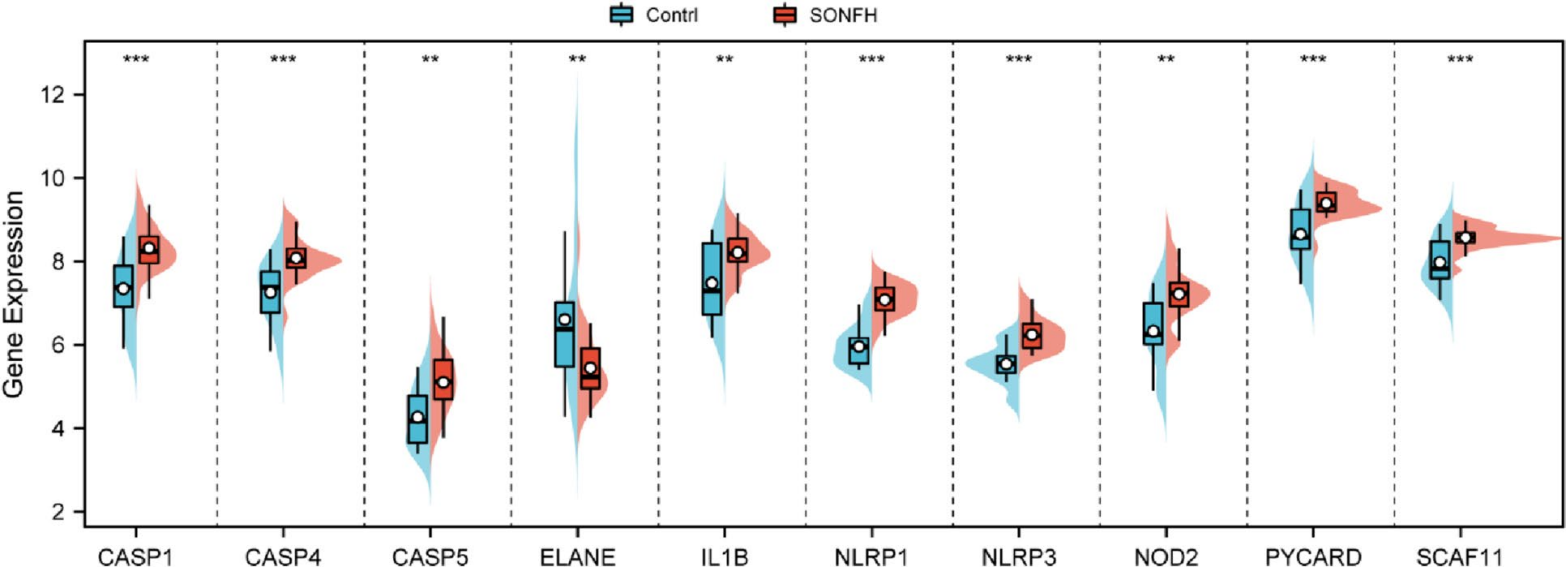
Bean plot of the 10 differentially expressed pyroptosis -related genes in SONFH and non-SONFH samples

The 10 pyroptosis-related differentially expressed genes of GSE123568 were presented as the volcano plots and the circo heatmap by the ggplot and pheatmap package R software in Fig. 1A and B. Immediately after, the expression patterns of 10 pyroptosis-related genes differentially expressed between SONFH and non-SONFH samples were shown in bean plot in Fig. 2.

### GO and KEGG enrichment analysis of pyroptosis-related differentially expressed genes

**Fig. 3:**
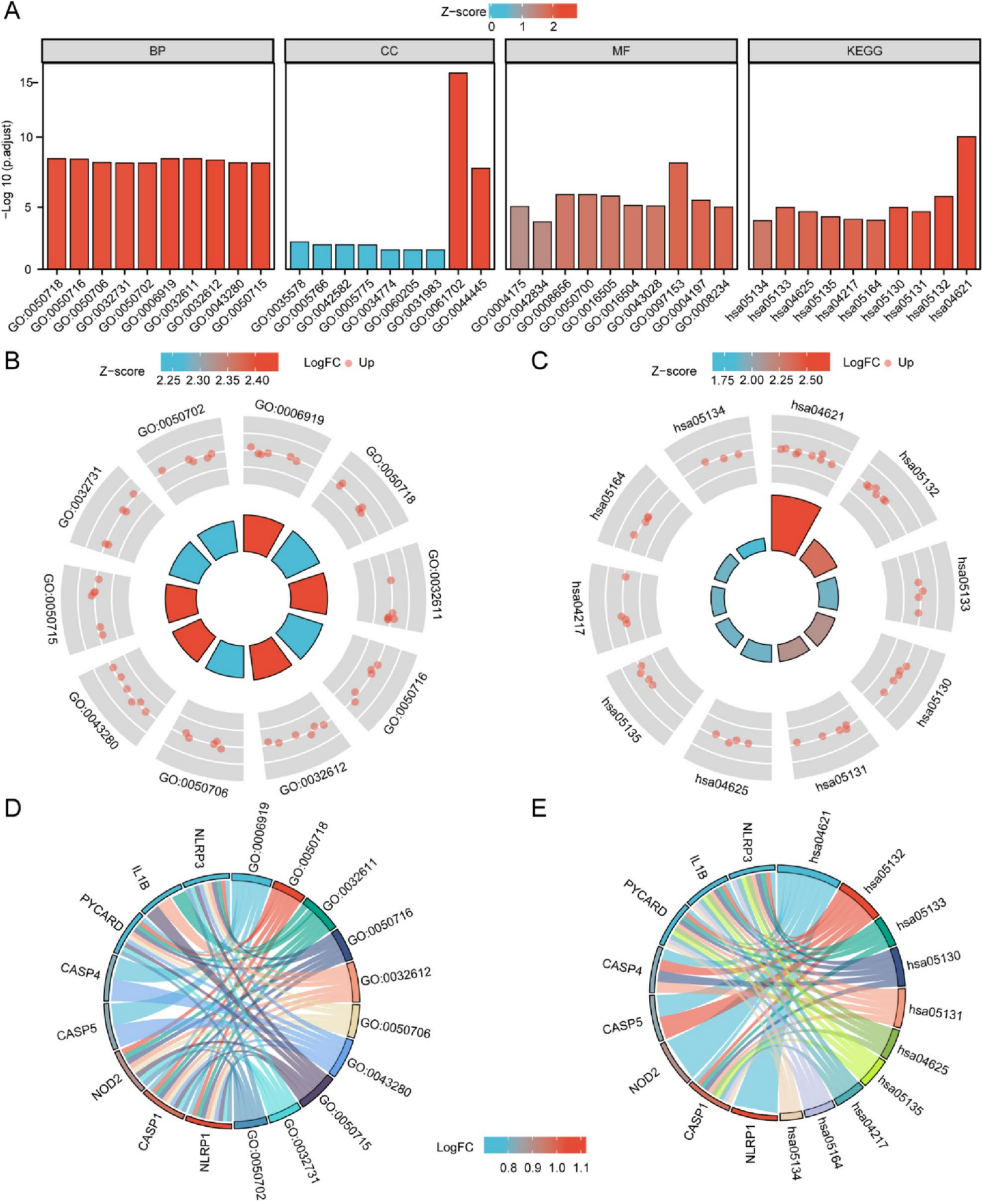
GO analysis and KEGG pathway functional enrichment analysis of 10 pyroptosis-related differentially expressed genes in SONFH and non-SONFH samples. **A** Bar plot of the top 10 enriched BP terms, CC terms MF terms and KEGG pathway terms of these 30 genes. **B** Circle plot of the top 10 enriched BP terms of these 10 genes. **C** Circle plot of top 10 enriched KEGG terms of these 10 genes. **D** Chord plot of relationship among the top 10 enriched BP terms and targets. **E** Chord plot of relationship among the top 10 enriched KEGG pathway terms and targets. The colours of the nodes range from red to blue in descending order of logFC values. The genes are ordered according to logFC values

We utilized the “clusterProfiler” R package to conducte GO and KEGG enrichment analyses of these pyroptosis-related differentially expressed genes. There were 481 significantly enriched BP terms, which were involved in activation of cysteine-type endopeptidase activity involved in apoptotic process, positive regulation of interleukin-1 beta secretion, interleukin-1 beta production, positive regulation of interleukin-1 secretion, and interleukin-1 production, as shown in Fig. 3A, B, D, and Table 2. A total of 9 significantly enriched CC terms were mainly correlated with inflammasome complex, cytosolic part, azurophil granule lumen, primary lysosome and azurophil granule, as shown in Fig. 3A. There were 22 significantly enriched MF terms including cysteine-type endopeptidase activity involved in apoptotic process, cysteine-type endopeptidase activator activity involved in apoptotic process, CARD domain binding, peptidase activator activity involved in apoptotic process and cysteine-type endopeptidase activity, as shown in Fig. 3A. In the KEGG enrichment analysis, these pyroptosis-related differentially expressed genes were significantly enriched in 13 KEGG pathway terms, such as NOD-like receptor signaling pathway, Salmonella infection, Pertussis, Pathogenic Escherichia coli infection and Shigellosis, as shown in Fig. 3A, C, E, and Table 2.

**Table 2:** The GO and KEGG Enrichment Analysis of 10 pyroptosis-related differentially expressed genes in SONFH samples compared to non-SONFH samples

ONTOLOGY	ID	Description	GeneRatio	BgRatio	pvalue	p.adjust	qvalue
BP	GO:0006919	activation of cysteine-type endopeptidase activity involved in apoptotic process	6/10	86/18670	1.66e-12	1.21e-09	4.06e-10
BP	GO:0050718	positive regulation of interleukin-1 beta secretion	5/10	33/18670	3.15e-12	1.21e-09	4.06e-10
BP	GO:0032611	interleukin-1 beta production	6/10	101/18670	4.45e-12	1.21e-09	4.06e-10
BP	GO:0050716	positive regulation of interleukin-1 secretion	5/10	38/18670	6.65e-12	1.36e-09	4.55e-10
BP	GO:0032612	interleukin-1 production	6/10	115/18670	9.85e-12	1.61e-09	5.39e-10
CC	GO:0061702	inflammasome complex	6/10	14/19717	7.72e-18	1.54e-16	6.50e-17
CC	GO:0044445	cytosolic part	6/10	247/19717	7.33e-10	7.33e-09	3.08e-09
CC	GO:0035578	azurophil granule lumen	2/10	91/19717	9.26e-04	0.006	0.003
CC	GO:0005766	primary lysosome	2/10	155/19717	0.003	0.011	0.004
CC	GO:0042582	azurophil granule	2/10	155/19717	0.003	0.011	0.004
MF	GO:0097153	cysteine-type endopeptidase activity involved in apoptotic process	4/10	15/17697	7.00e-11	2.73e-09	1.18e-09
MF	GO:0008656	cysteine-type endopeptidase activator activity involved in apoptotic process	3/10	16/17697	7.25e-08	9.42e-07	4.07e-07
MF	GO:0050700	CARD domain binding	3/10	16/17697	7.25e-08	9.42e-07	4.07e-07
MF	GO:0016505	peptidase activator activity involved in apoptotic process	3/10	19/17697	1.25e-07	1.22e-06	5.28e-07
MF	GO:0004197	cysteine-type endopeptidase activity	4/10	116/17697	3.57e-07	2.78e-06	1.20e-06
KEGG	hsa04621	NOD-like receptor signaling pathway	8/9	181/8076	4.82e-13	2.12e-11	1.32e-11
KEGG	hsa05132	Salmonella infection	6/9	249/8076	6.29e-08	1.38e-06	8.61e-07
KEGG	hsa05133	Pertussis	4/9	76/8076	8.81e-07	1.05e-05	6.54e-06
KEGG	hsa05130	Pathogenic Escherichia coli infection	5/9	197/8076	9.56e-07	1.05e-05	6.54e-06
KEGG	hsa05131	Shigellosis	5/9	246/8076	2.87e-06	2.28e-05	1.42e-05

### Correlation analysis of pyroptosis-related differentially expressed genes

**Fig. 4:**
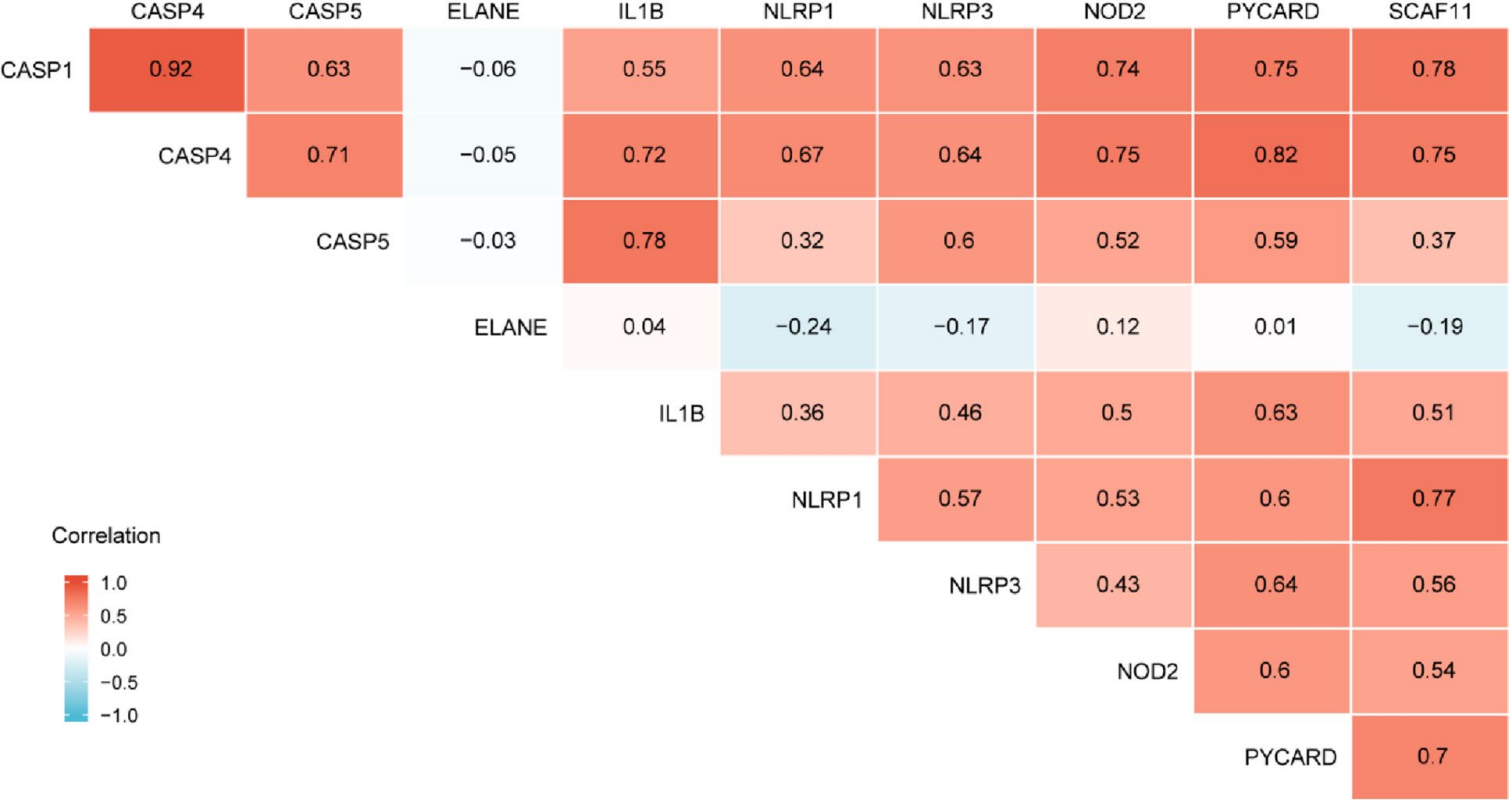
Spearman correlation analysis of the 10 differentially expressed pyroptosis-related genes in SONFH and non-SONFH samples

To explore the expression correlation among these pyroptosis-related differentially expressed genes in the GSE123568 dataset, we carried out the correlation analysis. The results suggested that some of these pyroptosis-related genes showed weak-to-moderate correlations. Among these 10 genes, CASP1 and CASP4 were most positively correlated (Cor = 0.92), while NLRP1 and ELANE (Cor =  − 0.24) were most negatively correlated as shown in Fig. 4.

### PPI network of pyroptosis-related differentially expressed genes

**Fig. 5:**
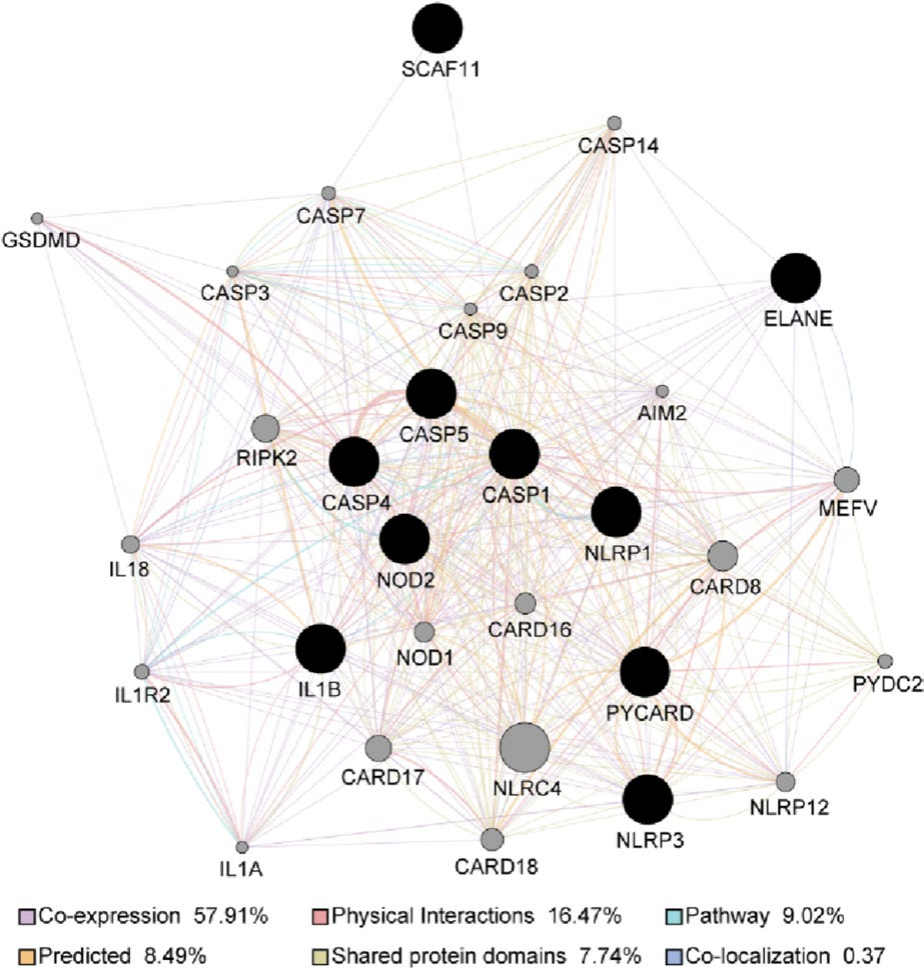
PPI network of 10 pyroptosis-related differentially expressed genes in SONFH and non-SONFH samples. Black nodes represent target proteins, and connecting colors indicate different correlations. Functional associations between targets were investigated using GeneMANIA. Genes in black circles were query terms while these in gray circle indicate genes associated with query genes

We conducted a PPI analysis by using the GeneMANIA database, to determine the in-depth interactions of these pyroptosis-related differentially expressed genes. As shown in Fig. 5, the results of GeneMANIA also revealed that the functions of these pyroptosis-related genes interacted with each other and the number of interactions of each gene, which was found that 57.91% had co-expression, 16.47% physical interactions, 9.02% pathway, 8.49% predicted, 7.74% shared protein domians, and 0.37% co-localization.

### ROC curve analysis of pyroptosis-related differentially expressed genes

**Fig. 6:**
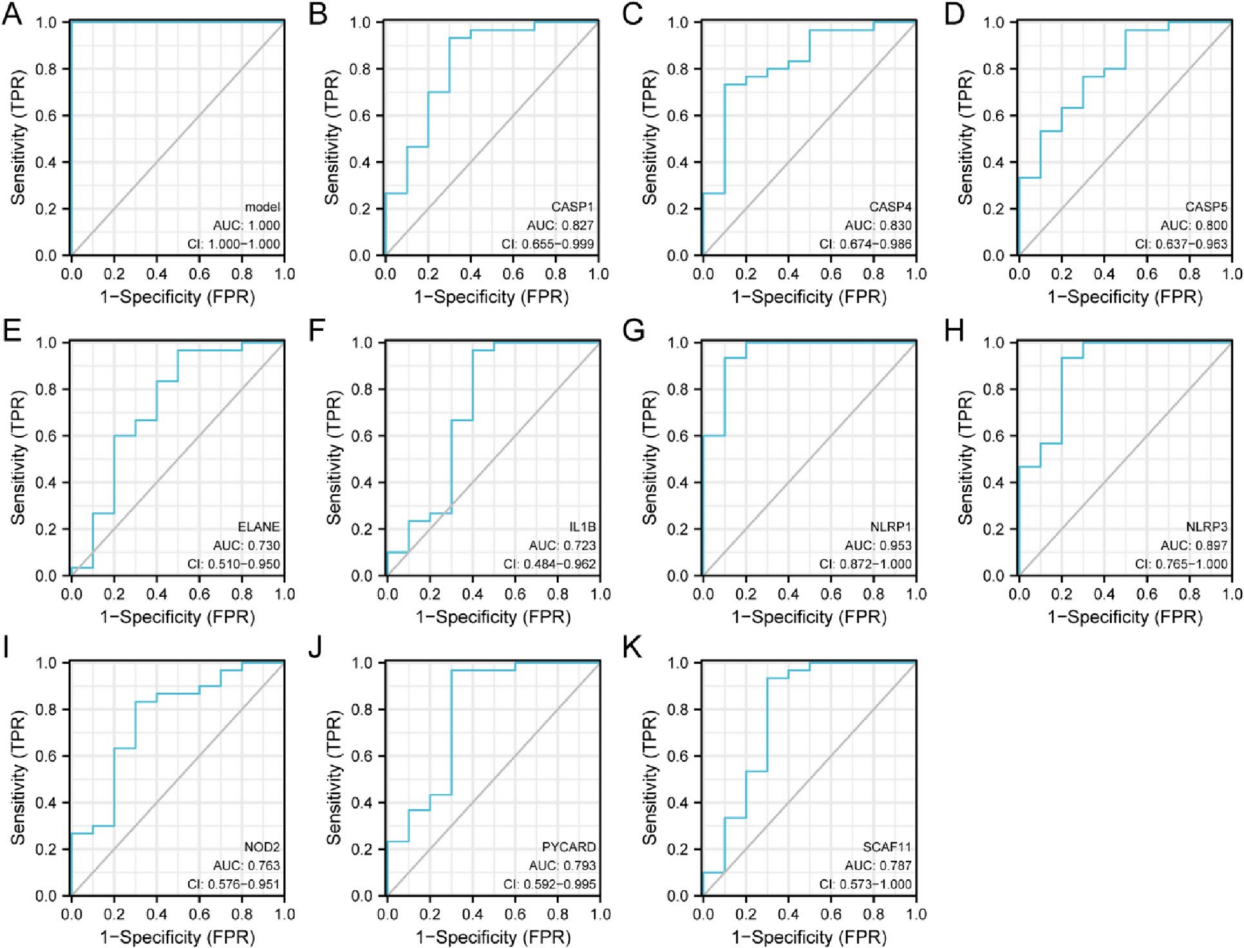
ROC curve of the 10 pyroptosis-related differentially expressed genes in SONFH samples

The ROC curve analysis of these pyroptosis-related differentially expressed genes of SONFH samples were analyzed, established and drawn by “pROC” and “ggplot2” R package. Area under the curve (AUC) values indicated an indicator combining sensitivity and specificity metrics, which could describe the intrinsic effectiveness of diagnostic tests. Compared to non-SONFH samples, these pyroptosis-related differentially expressed genes had good predictive diagnostic efficacy (AUC = 1.000, CI = 1.000–1.000) in the SONFH samples as shown in Fig. 6A, which might be potential pyroptosis-related biomarkers for SONFH based on our present samples. Among them, NLRP1 had the highest diagnostic value (AUC: 0.953) in the SONFH samples. The diagnostic values of other genes were also performed as follows in SONFH samples as shown in Fig. 6B-K: NLRP3 (AUC: 0.897), CASP4 (AUC:0.830), CASP1 (AUC:0.827), CASP5 (AUC:0.800), PYCARD (AUC: 0.793), SCAF11 (AUC: 0.787), NOD2 (AUC: 0.763), ELANE (AUC: 0.730) and IL1B (AUC:0.723).

## Discussion

The most common cause of non-traumatic osteonecrosis of the femoral head is alcohol and glucocorticoids [22]. Since Pietrogrande et al. reported the first case of SONFH [23], glucocorticoids as one of the pathogenic factors have been shown to be directly associated with the onset of SONFH [24]. With the wide application of glucocorticoids in clinical practice, the incidence of SONFH has increased year by year, which has surpassed alcoholic osteonecrosis to become the first major cause of non-traumatic osteonecrosis [25]. Through a follow-up study, Li Zirong et al. found that most SONFH occurred within 2 years after medication, mainly within 6 months. Moreover, high-dose glucocorticoids shock therapy and long-term application of glucocorticoids were closely related to SONFH, while the short-term application of low-dose glucocorticoids had no obvious correlation with SONFH [25–28]. Patients in the early stage of SONFH did not have any clear symptoms, and the symptoms cannot be reversed, and the incidence rate increased year by year [29]. But some studies have shown that under the action of glucocorticoid, normal connection mechanism, signal transmission, osteocytes, osteoblasts cannot according to the external stimulus orderly repair, reshape the normal structure, the mechanical properties of the femoral head cannot effectively reconstruct, and cannot restore the normal femoral head mechanical support structure, and exhibit local sclerosis cystic changes, and even collapse [30–34]. Therefore, the early and accurate timing of hip-preserving treatment is the key to the success treatment of SONFH [25].

Although glucocorticoids have been recognized as the pathological cause of SONFH, the exact mechanisms underlying their specific pathogenesis and progression are not fully understood, which was a dynamic pathological process jointly regulated by multi-target, multi-pathway, multi-link, and multi-stage cooperation, involving the interaction of various molecular and biological processes [35]. With the further deepening of basic and clinical research of SONFH, the increasingly highly recognized pathogenic mechanisms were proposed to explain the pathogenic mechanisms, including intravascular coagulation, uncoupled bone remodeling, lipid metabolism, abnormal differentiation of MSCs, oxidative stress injury, programmed cell death, and so on [36]. Among them, changes in osteocyte cell activity due to programmed cell death were the intermediate link in various theories. Programmed cell death was an active process controlled by relevant genes that regulates body development and maintains a stable internal environment [37]. Many scholars believe that programmed cell death may play a key role in the process of SONFH. Pyroptosis was a programmed cell necrosis different from apoptosis and autophagic inflammation and proposed by Boise in 2001, which is a natural immune barrier for the human body [7]. The morphological aspect of pyroptosis is characterized by swelling of cells, formation of pyroptotic bodies, membrane rupture and releasing inflammatory factors [38]. At present, it has been confirmed that the extensive use of GC could activate the pyroptosis of BMSCs, which is closely related to the occurrence of SONFH [15]. It was found that high Nlrp^3D301N^ expression causes bone mass loss, mainly due to the role of inflammatory factors such as IL-1β, facilitated by NLRP3 in mouse models specifically expressing Nlrp^3D301N^ in osteoclasts, to further activate osteoclasts to cause bone resorption [39]. Liu et al. demonstrated the presence of pyroptosis from the perspective of LPS inducing oxidative stress in osteoblasts, and the inhibition of pyroptosis enhances osteoblast differentiation and activity [40]. Glucocorticoids on the signal regulation of programmed cell death, especially cell pyroptosis, was a complex process, cells in different states of glucocorticoid effect, under a certain concentration of glucocorticoids could effectively promote bone tissue related cell proliferation activity, rebuild normal signaling network. Tao et al. indicated that pyroptosis was proposed as a novel hypothesis of bone-related diseases pathogenesis for the first time, thus providing a new direction for the treatment of bone-related diseases in the future [41]. Therefore, this study focuses on the expression level changes of key pyroptosis-related genes in SONFH, and the achievement of tone results will have important theoretical and practical significance for the pathological mechanism, early diagnosis and even prognosis of SONFH.

To the best of our knowledge, no relevant articles have yet identified pyroptosis-related genes in SONFH based on bioinformatics analysis and animal experimental validation so far. There were 10 pyroptosis-related differentially expressed genes associated with SONFH based on the intersection of the 33 non-duplicated pyroptosis-related genes referring to the article of Ye et al. and differentially expressed genes from the GSE123568 dataset according to the defined criteria of an adjusted *P*-value < 0.05 and |logFC|> log_2_1.5, including 9 up-regulated genes and 1 down-regulated genes. The 10 pyroptosis-related differentially expressed genes in SONFH samples compared to non-SONFH samples included CASP1, CASP4, CASP5, ELANE, IL1B, NLRP1, NLRP3, NOD2, PYCARD and SCAF11.

To further elucidate the underlying mechanism of the 10 pyroptosis-related differentially expressed genes in SONFH, we evaluated their biological functions and signaling pathways GO and KEGG enrichment analysis by the “clusterProfiler” R package. The GO and KEGG enrichment analyses showed that the 481 BP terms, the 9 CC terms, the 22 MF terms and 13 KEGG terms were significantly enriched, such as cysteine-type endopeptidase activity involved in apoptotic process, positive regulation of interleukin-1 beta secretion, and NOD-like receptor signaling pathway, and so on. The 10 genes involved in pyroptosis were further carried out by the correlation analysis, and the result showed CASP1 and CASP4 were most positively correlated, while NLRP1 and ELANE were most negatively correlated. At the same time, we constructed the PPI network on the above differentially expressed genes using the GeneMANIA database. The PPI networks might consist of several functional modules, which had co-expression (57.91%), physical interactions (16.47%), pathway(9.02%), predicted(8.49%), shared protein domians(7.74%) and co-localization(0.37%). Besides, compared to non-SONFH samples, the diagnostic values of these above 10 genes were performed as follows: NLRP1, NLRP3, CASP4, CASP1, CASP5, PYCARD, SCAF11, NOD2, ELANE and IL1B; and these 10 pyroptosis-related differentially expressed genes had higher diagnostic value (AUC = 1.000, CI = 1.000–1.000) in the SONFH samples, which might be potential pyroptosis-related biomarkers for SONFH.

Some of these SONFH and pyroptosis-related genes have been previously studied. For example, Yu and his colleagues showed that IL1B gene influences the genetic susceptibility of SONFH [42] Li et al. identified that NOD2 might be potential inflammation-related biomarkers of SONFH by weighted gene co-expression network analysis (WGCNA) and inflammation-related genes (IRGs) from the Molecular Signatures Database (MSigDB) [43]. Further studies are needed to reveal the roles and mechanisms of potential pyroptosis-related differentially expressed genes in SONFH.

Although the pyroptosis-related genes potentially involved in SONFH have been mined via bioinformatics and validation of animal experiments, there were still some limitations in this study. First, although there was a large amount of high-throughput data stored in the GEO database, we still did not find more *homo spapiens* datasets about SONFH, which led to a particularly limited number of samples. Second, further experiments of human blood specimens are lacking to pyroptosis-related validate the differentially expressed genes and their accuracy and specificity as diagnostic biomarkers. Third, the specific their underlying molecular mechanisms of differential genes in the pathogenesis and progression of SONFH are still unclear, and more basic experiments were needed to elaborate. Therefore, further research is necessary to address the possible limitations in terms of biased results and conclusions.

## Conclusion

In conclusion, there were 10 potential pyroptosis-related differentially expressed genes involved in SONFH identified based on bioinformatic analysis in the present study, which might serve as potential diagnostic biomarkers of pyroptosis after SONFH. These results would help to further explore the development of SONFH by regulating pyroptosis, and to provide data support and new research ideas for the diagnosis of SONFH.

## Data Availability

Publicly available datasets were analysed in this study. These datasets can be found at the following URLs: GSE123568 (https://www.ncbi.nlm.nih.gov/geo/query/acc.cgi?acc=GSE123568).

## Abbreviations

AUC: Area Under the Curve
BP: Biological Process
BMSC: Bone Marrow Mesenchymal Stem Cells
CC: Cellular Component
FC: Fold Change
GC: Glucocorticoids
GEO: Gene Expression Omnibus
GO: Gene Ontology
KEGG: Kyoto Encyclopedia of Genes and Genomes
LPSL: Lipopolysaccharide
MF: Molecular Function
NCBI: National Center for Biotechnology Information
PPI: Protein–Protein Interaction
SD: Sprague–Dawley
SONFH: Steroid-Induced Osteonecrosis of the Femoral Head
